# Hospital Meetings, &c.

**Published:** 1897-05-08

**Authors:** 


### HOSPITAL MEETINGS, &C.

### ZENANA BIBLE AND MEDICAL MISSION.

THE annual meeting of the Zenana Bible and Medical Mission was held at St. Martin's Town Hall on Wednesday, April 28th. Lord KINNAIRD was in the chair, and amongst others present were Lord Radstock, the Hon. Gertrude Kinnaird, and Dr. Cust. The speakers were Miss Clifford, Miss Wilder, and the Rev. H. C. Squires. The society has at present 34 centres of work in India, employing 129 European missionaries and assistants, 191 native Christian teachers, nurses, &c., and 78 Bible women. The missionaries and Bible women have access to 7,988 Zenanas and houses. The society's work among the sick has developed considerably during the past year. A new wing has been added to the Lady Kinnaird Memorial Hospital at Lucknow, raising the accommodation to 50 beds for in-patients, and providing an isolation ward for infectious cases. At Lucknow, in addition to two fully-qualified lady doctors, there is a qualified native Christian doctor, an English nurse to train the native women, and a missionary; and a new dispensary was opened in a neighbouring small town last year. At Benares and at Patna the work has grown rapidly, and the new hospital at the latter place has proved a great boon. The pressing need for increased funds is causing the society much anxiety, the present year of famine and plague in India involving much additional expenditure, and the committee are making special efforts to raise the income of the society to £25,000. The total income from all sources during 1896 amounted to £18,067 2s. 1d., and the accounts show a deficiency of about £2,000. Contributions will be thankfully received by the treasurers (Lord Kinnaird, 1, Pall Mall East, S.W., and Sir William Muir, K.C.S.I., Dean Park House, Edinburgh).

